# How to specify healthcare process improvements collaboratively using rapid, remote consensus-building: a framework and a case study of its application

**DOI:** 10.1186/s12874-021-01288-9

**Published:** 2021-05-11

**Authors:** Jan W. van der Scheer, Matthew Woodward, Akbar Ansari, Tim Draycott, Cathy Winter, Graham Martin, Karolina Kuberska, Natalie Richards, Ruth Kern, Mary Dixon-Woods, Thiscovery Authorship Group, André Sartori, Andy Paterson, Doro Unger-Lee, Joann Leeding, Luke Steer, Obstetric Emergency Consensus Authorship Group, Amanda Andrews, Rita Arya, Sarah F. Bell, Denise Chaffer, Andrew Cooney, Rachel Corry, Mair G. P. Davies, Lisa Duffy, Caroline Everden, Theresa Fitzpatrick, Courtney Grant, Mark Hellaby, Tracey A. Herlihey, Sue Hignett, Sarah Hookes, Fran R. Ives, Gyuchan T. Jun, Owen J. Marsh, Tanya R. Matthews, Celine McKeown, Alexandra Merriman, Giulia Miles, Susan Millward, Neil Muchatata, David Newton, Valerie G. Noble, Pamela Page, Vincent Pargade, Sharon P. Pickering, Laura Pickup, Dale Richards, Cerys Scarr, Jyoti Sidhu, James Stevenson, Ben Tipney, Stephen Tipper, Jo Wailling, Susan P. Whalley-Lloyd, Christian Wilhelm, Juliet J. Wood

**Affiliations:** 1 THIS Institute (The Healthcare Improvement Studies Institute), Department of Public Health and Primary Care, University of Cambridge, Cambridge Biomedical Campus, Clifford Allbutt Building, Cambridge, CB2 0AH UK; 2 Department of Translational Health Services, University of Bristol, Bristol, UK; 3 PROMPT Maternity Foundation, Women and Children’s Health, North Bristol NHS Trust, Westbury on Trym, UK

**Keywords:** Consensus-building, Consensus development, Delphi technique, Best practices, Professional practice, Obstetrics, Postpartum haemorrhage, COVID-19

## Abstract

**Background:**

Practical methods for facilitating process improvement are needed to support high quality, safe care. How best to specify (identify and define) process improvements – the changes that need to be made in a healthcare process – remains a key question. Methods for doing so collaboratively, rapidly and remotely offer much potential, but are under-developed. We propose an approach for engaging diverse stakeholders remotely in a consensus-building exercise to help specify improvements in a healthcare process, and we illustrate the approach in a case study.

**Methods:**

Organised in a five-step framework, our proposed approach is informed by a participatory ethos, crowdsourcing and consensus-building methods: (1) define scope and objective of the process improvement; (2) produce a draft or prototype of the proposed process improvement specification; (3) identify participant recruitment strategy; (4) design and conduct a remote consensus-building exercise; (5) produce a final specification of the process improvement in light of learning from the exercise. We tested the approach in a case study that sought to specify process improvements for the management of obstetric emergencies during the COVID-19 pandemic. We used a brief video showing a process for managing a post-partum haemorrhage in women with COVID-19 to elicit recommendations on how the process could be improved. Two Delphi rounds were then conducted to reach consensus.

**Results:**

We gathered views from 105 participants, with a background in maternity care (*n* = 36), infection prevention and control (*n* = 17), or human factors (*n* = 52). The participants initially generated 818 recommendations for how to improve the process illustrated in the video, which we synthesised into a set of 22 recommendations. The consensus-building exercise yielded a final set of 16 recommendations. These were used to inform the specification of process improvements for managing the obstetric emergency and develop supporting resources, including an updated video.

**Conclusions:**

The proposed methodological approach enabled the expertise and ingenuity of diverse stakeholders to be captured and mobilised to specify process improvements in an area of pressing service need. This approach has the potential to address current challenges in process improvement, but will require further evaluation.

**Supplementary Information:**

The online version contains supplementary material available at 10.1186/s12874-021-01288-9.

## Background

The last three decades have seen clinical guidelines, defined as “systematically developed statements informed by a systematic review of evidence and an assessment of the benefits and harms of care options designed to optimize patient care” [1], become a cornerstone of evidence-based practice. Production of guidelines is based on well-established methodologies, including synthesis of scientific evidence, expert opinion, and stakeholder consultation, and is supported by an infrastructure of national and international bodies (e.g. government agencies and professional associations) [2]. Clinical guidelines are not, of course, self-implementing. Getting clinical guidance into practice is typically complex, requiring multi-modal approaches, and is the subject of a burgeoning science and associated literature [3, 4]. It is now clear, however, that the implementation of evidence-based practices (*what* should be done) depends crucially on process improvement – changes to *how* things are done [5, 6]. For this reason, the specification of process improvements (i.e. identifying and defining the changes in processes that need to be made to deliver good care) is a key task. In this article, we offer an approach for developing specifications for process improvements using rapid, remote, consensus-building methods, and we illustrate it using a case study conducted during the COVID-19 pandemic.

We start by noting that there is no consensual definition of process improvement, but it is distinguished by its focus on how to improve the underlying processes (such as workflows, task design, role allocations, communication techniques, resources required, and so on) for delivering care – rather than, as in the case of clinical guidelines, defining ideal clinical standards. Process improvement has a particular role in ensuring that work systems are optimised, for example by helping to define the activities from beginning to end of a clinical process or pathway; to explain how these activities can most effectively be undertaken; to clarify tasks, roles, and skills needed; to characterise the decisions to be made and the support needed to make and implement those decisions; and to identify the equipment, resources and other tools required [7, 8].

The various methods for process improvement are often gathered together under the rubric of *quality improvement* [9, 10]. Some, such as the Model for Improvement, Lean and Six Sigma, amongst others [11], have been adapted from industry techniques [12]. Other approaches have been developed from the design and engineering disciplines and draw on socio-technical systems principles. For example, human factors and systems engineering use structured methods to change existing work systems based on the analysis of the interactions between people, tasks, tools, technology and the environment [13–15].

Common to most of these approaches is the need to *specify* process improvements. Specification requires identifying and defining the changes that need to be made (for example, an amendment to task or role design, or use of a new piece of equipment) to bring about improvement. A major challenge, however, is that the large-scale infrastructure for developing specifications for process improvement in healthcare has, in contrast with clinical guideline development, remained under-developed. The work of process improvement specification has instead remained largely locally-led, conducted within individual organisations.

Local leadership of process improvement has, of course, many advantages, including the potential to customise a solution to local circumstances and to imbue a sense of local ownership, but it is also associated with some disadvantages. One is that each individual organisation facing circumstances requiring change may develop their own process improvement specification in isolation [16, 17], but a multiplicity of approaches to the same area of practice may cause problems of its own. First, it may be wasteful, with each organisation reinventing the wheel [17, 18]. Second, it may be sub-optimal: reaching the best possible solution requires inputs from multiple disciplines, but individual organisations may lack access to the fullest range of expertise, particularly when it is rare (e.g. specialist human factors knowledge) [19–21]. Third, destandardisation creates learning overheads and new risks, for example when personnel moving between institutions have to learn new ways of doing things while unlearning previous ones. But while exclusively bottom-up development of ways of working may not be ideal, top-down imposition of particular practices can generate other pathologies [22], including failures of implementation, truculent and dysfunctional compliance, inadequate customisation to local circumstances, and creation of perverse incentives.

The deficiencies in some of the current infrastructure for process improvement have been vividly surfaced by the COVID-19 pandemic, which has caused massive disruption in the organisation and delivery of healthcare [23, 24]. Rapid innovation has been a feature of the response, prompted by the need to make adaptations to established clinical processes to address the infection risks and other challenges associated with the virus, as well as many other changes to clinical pathways and practices [25–35]. While the scale and speed of response to the pandemic has been impressive, an important risk is that some efforts may unhelpfully reproduce some of the challenges previously identified in the field of quality improvement [17, 36], including those discussed above of duplication of effort, waste, de-standardisation, and inability to engage sufficiently diverse expertise.

Given the challenges of both top-down and bottom-up quality improvement, a more collaborative alternative is likely to be of value. In this article, we address this need. We propose a methodological approach designed to enable large-scale remote engagement and mobilisation of multiple forms of expertise to build rapid consensus on specifications of process improvements, and we describe a case study of its application.

## Methods

In developing our approach, we built on a participatory ethos, principles of crowd-sourcing, and consensus-building methods.

### Participatory ethos

A participatory ethos – an approach that values the perspectives of the full range of groups of people affected by an issue – is an important guiding value in healthcare improvement [37]. But it is also of practical significance: securing participation may be more likely to result in solutions that are satisfying, workable, informed by professional values and clinical expertise, capable of being customised for specific situations, and capable of being implemented through collective effort rather than harsh, externally-imposed sanctions [38, 39]. Participatory approaches may also be more likely to lead to sustainable impacts by generating a sense of local ownership and commitment [40, 41]. Participatory approaches may be especially useful in encouraging practitioners to engage with evidence and its creation [42], as well as generating findings that have impact on practice [43].

### Crowdsourcing

By drawing on the collective intelligence of many individuals, crowdsourcing can enable data to be collated on a much greater scale than would otherwise be possible [44], creating potential to solve problems by drawing on a wider range of perspectives and diverse experiences and knowledge [45]. Recent advances such as online engagement platforms [46–48] are now facilitating engagement of large, diverse and geographically dispersed stakeholders remotely as collaborators in co-constructing solutions [44, 49–51].

### Consensus-building methods

Consensus-building methods are well established as ways of promoting deliberation, inclusion, and participation in situations where there may be multiple perspectives, interests and communities [52–56]. Methods widely used in developing guidance in healthcare include the nominal group technique [57, 58], the consensus development conference [59, 60], the RAND/UCLA appropriateness method [61, 62], and the Delphi method [63, 64]. These approaches are commonly recommended and used for developing clinical guidelines [55, 65] and reporting guidelines [66, 67], but application of consensus-building in process improvement has remained much more limited, not least because of the tendency (discussed above) to see process improvement as the domain of local teams. Yet consensus-building is potentially of value for process improvement in helping to build shared understanding, to include diverse forms of expertise, and to produce agreements about process improvement that might otherwise remain elusive.

The Delphi method offers considerable promise in this respect, as one of the best known and most widely used approaches for consensus-building in healthcare contexts [52, 54]. Using group communication that brings together and synthesises knowledge, participants are typically involved in a number of rounds of rating or voting on a set of propositions, and may then adjust their initial ratings based on feedback from the group in a number of subsequent iterations [68, 69]. Though Delphi can include a large number of individuals across diverse locations and areas of expertise [54], many Delphi exercises for healthcare have only involved relatively small and homogeneous panels of approximately 10 to 30 participants [70]. There is evident scope for including larger and diverse groups of participants [54, 70]. New methodological approaches are needed to use the method effectively for large-scale remote consensus-building to specify process improvements, while adhering to a participatory ethos and minimising time and effort required of participants.

### A proposed methodological approach for developing specifications for process improvements using rapid, remote, consensus-building methods

The approach we propose for rapid consensus-building of process improvement specifications involves five steps, which we have organised into a framework (Table 1). The steps are: (1) define scope and objective of the process improvement; (2) produce a draft or prototype of the proposed process improvement specification; (3) identify participant recruitment strategy; (4) design and conduct a remote consensus-building exercise; (5) produce a final specification of the process improvements in light of learning from the exercise.

**Table 1:** Framework for rapid, participatory, remote consensus-building for process improvement specification

**1) Define scope and objective of the process improvement**	Identify and characterise the problem to be solved Assess the extent to which consensus-building is an appropriate method for the problem to be solved Define objective for the project Define target audiences for output of the project
**2) Produce draft or prototype of the proposed process improvement specification**	To inform the draft/prototype, use rapid literature reviews, existing guidelines, ideas sourced from specialist groups, or stakeholder surveys, interviews and focus groups Create a resource reflecting the draft or prototype with the proposed process improvement specification (e.g. video-based simulated scenario, standard operating procedure, piece of equipment)
**3) Identify participant recruitment strategy**	Identify and select stakeholder groups using principles of relevance, inclusion and diversity Define strategies for ethics, recruitment, sampling and sample size
**4) Design and conduct a remote consensus-building exercise**	Ethics Data collection and analysis
**5) Produce a final specification of the process improvements in light of learning from the exercise**	Create resources that reflect the specified process improvements informed by the draft/prototype and the consensus-building exercise Disseminate the resources

#### (1) Define scope and objective of the process improvement

A first step is to identify and characterise the problem to be solved and the goal to be achieved so that an assessment can be made of whether consensus-building is an appropriate method [71]. If consensus-building is selected, the next step is to define the scope and objective of the process improvement, similar to current practices for developing clinical practice guidelines [72] and Core Outcome Sets [54]. These activities require close collaboration with key stakeholders, in accordance with the participatory principle [37, 73, 74].

#### (2) Produce a draft or prototype of the proposed process improvement specification

Rapid consensus-building on the specification of process improvements can be facilitated by producing a draft or prototype version, which might be informed by rapid literature reviews, existing guidelines or practices, ideas sourced from specialist groups, or stakeholder surveys, interviews and focus groups [70, 75]. Feedback can then be sought on this draft/prototype, informing subsequent rounds of Delphi consensus-building. The draft/prototype may take a range of forms [75], for example a conceptual framework, an existing standard operating procedure, a video-based simulated scenario, or an artefact (e.g. equipment or software). The most appropriate form can be selected based on the technical aspects of the healthcare process, alignment with the goals of the final product, expectations of participants’ available time, questions of how to maximise participant engagement, and possibilities and limitations of (mobile) devices and platforms on which the draft/prototype will be presented [76, 77].

#### (3) Identify participant recruitment strategy

##### Eligibility

Determining participant eligibility criteria requires consideration of the need for triangulation and bringing together the views of different types of stakeholders [54, 55, 78–80]. Seeking diversity can reduce risk of bias and provide a richer variety of views [78, 81–83]. Eligibility criteria might include specialists working in the selected field of clinical practice (e.g. maternity staff) or those with specialist expertise (e.g. infection prevention, human factors). Importantly, for many process improvement activities, patients and the public are key stakeholders whose expertise and perspectives should be included [42, 84].

##### Sample size

The current literature provides no set standards for required sample sizes for Delphi exercises, meaning that pragmatic choices have to be made [54, 85]. One approach is to estimate the sample size based on the number that would likely result in stable ratings across the Delphi rounds, accommodating for dropout [85, 86]. This may require establishing a minimal sample size for each stakeholder group included.

##### Sampling and recruitment

Sampling strategies for consensus-building exercises are generally informed by available time and resources, and include convenience, purposive or criterion sampling [71, 85]. Recruitment strategies can, for example, be grounded in the voluntary contribution of willing individuals who wish to contribute to the production of scientific knowledge [87–89]. The principles of participatory research may inform successful strategies for increasing participation of minority groups [89, 90].

##### Retention

Measures to support retention across the different phases of the consensus-building exercise may involve strategies such as e-mail reminders [91] that include reference to the importance of the participant’s contributions [87]. This could be enhanced by the use of online engagement platforms [46–48], which may help encourage feelings of being part of a research community.

#### (4) Design and conduct a remote consensus-building exercise

##### Ethics

The ethical principles for participatory consensus-building exercises have much in common with any other quality improvement activity [92, 93], or indeed guideline development, in that they may often be classified as service improvement activities not requiring specific ethical review. Where these exercises are conducted as research studies rather than improvement activities, different considerations may apply, such as the requirement for oversight and/or approval by an Institutional Review Board or Research Ethics Committee [92, 93].

##### Idea generation

Delphi rounds are typically informed by an exercise to generate ideas [63, 64]. This might be done by inviting participant feedback on the draft/prototype of the process improvement specification, which can then be synthesised in a set of propositions to be rated in the subsequent Delphi rounds. If this approach is taken, using open-ended, short-answer options may work best for a rapid response with minimal burden for participants and analysts [94]. The synthesis method depends on the type of feedback generated, and needs to be clearly documented [75]. Synthesis might involve, for example, removal of duplicates and merging of responses with similar wording [95]; using thematic analysis to create concepts, categories or themes [96]; or coding individual responses to themes and triangulating the coding among multiple researchers [95, 97]. The aim is to balance the consolidation of responses (i.e. creating a list that is reasonable for experts to navigate in subsequent rounds, e.g. about 20 items [94, 95]) while avoiding excessive abstraction [97].

##### Delphi rounds

The propositions derived from the idea generation phase can be used in two or more subsequent Delphi rounds of iterative surveys in which participants state their level of agreement with propositions on a numeric scale. Responses are aggregated and participants have the opportunity to revise their judgments in the light of feedback that includes their own and the group’s judgment, with the aim of exploring or reaching group consensus [98]. Two rounds is often sufficient to reach consensus and may reduce burden for participants [70, 75], though more rounds may sometimes be used. Use of visually appealing forms of feedback, such as interactive graphs that show the distributions of ratings across one or more stakeholder groups, can facilitate response across rounds.

#### (5) Produce a final specification of the process improvements in light of learning from the exercise

The aim of the Delphi exercise is to produce recommendations that can inform the specification of process improvements that can be implemented at scale. A plan should be in place for developing and disseminating supporting resources that reflect the specified process improvements. One advantage of the collaborative approach is that it is likely to facilitate engaged dissemination and implementation by those who have participated [99].

### Case study

We tested the proposed methodological framework using an example from emergency maternity care. The need for process improvement specification in maternity care during the COVID-19 crisis was particularly urgent given that, in contrast with some other areas of care, it is not possible to defer or reschedule births [100, 101]. During the first few months of the pandemic [23, 24], approximately 100 pregnant women with suspected or confirmed COVID-19 were admitted each week to obstetric units across the UK [102]. This meant that many existing areas of care where good clinical practice was well-established (e.g. through clinical guidelines) required process improvement to adapt to the need for: infection control, the challenges of communication and teamwork likely to be posed by use of personal protective equipment (PPE), and other demands of making clinical processes COVID-safe. It was important, for example, that maternity staff were able to adapt quickly to the new infection prevention requirements of donning PPE to minimise any delays to providing prompt clinical treatment.

The result was that maternity units were all, individually, urgently seeking COVID-19-specific resources and training for obstetric emergencies. Responding to the emerging but still limited guidance for dealing with COVID-19 in maternity care (e.g. [103]), NHS maternity professionals expressed a need for clearer e-learning resources relating to PPE skills, and more training on COVID-19-specific emergency drills [104]. One priority area concerned how to manage an obstetric emergency such as post-partum haemorrhage (PPH) in a woman with suspected or confirmed COVID-19. PPH is an emergency that complicates 1.2% of births in high-income settings [105, 106]. It is one of the most frequent and severe maternal complications after birth [107–109], and a cause of intensive care admission in the UK [110]. It is a classic example of where the requirements for managing situation clinically are well understood and communicated through clinical guidelines (e.g. [111, 112]), but where process improvement was needed to ensure that the underlying processes of delivering care were adapted to deal with the challenges imposed by the pandemic.

Addressing this problem required rapid, remote consensus-building among multi-professional stakeholder groups to specify process improvements for managing an obstetric emergency in a woman with suspected or confirmed COVID-19. To employ a consensus-building exercise, we used Thiscovery (https://www.thiscovery.org/about), an online research and development platform created and developed by THIS Institute at the University of Cambridge. One of its founding goals is that of facilitating inclusive, multi-stakeholder involvement while offering maximum flexibility and minimum burden.

## Results

We applied the five steps of the framework to the case study. We: (1) defined the scope and objective of the project with key stakeholders; (2) produced a draft video showing a simulation of processes for handling an obstetric emergency in a COVID scenario; (3) recruited three expert groups (maternity care, infection prevention and control, and human factors specialists); (4) designed and conducted an exercise to reach consensus on recommendations to improve the processes illustrated in the video; and (5) produced a final specification of the process improvement, informed by the consensus-built recommendations, and illustrated this specification in an updated video and other resources .

### (1) Scope and objective

The project had its origins early in the pandemic when key stakeholder organisations, including the Royal College of Midwives and Royal College of Obstetricians and Gynaecologists, identified that while clinical guidance on how to handle obstetric emergencies such as PPH remained sound, the underlying processes required adaptation for women with confirmed or suspected COVID-19. Many maternal deaths related to PPH in healthcare settings can be avoided through effective clinical management [106, 109], including prompt initiation of several simultaneous actions such as uterine massage, intravenous fluid resuscitation, and administration of medication (tranexamic acid to treat major haemorrhage and uterotonics to contract the uterus). Treatment delay can result in poor outcomes [113, 114], so delivering these clinical interventions requires highly optimised underlying processes, including effective teamwork, coordination, communication, and access to appropriate supplies. All of these processes require adaptation for a COVID-19 scenario, which might demand, for example, donning and doffing of personal protective equipment, changes in the tasks undertaken and their sequencing, and forms of communication suitable for a situation where masks and visors may inhibit verbal and non-verbal exchange.

This problem appeared well-suited to consensus-building that could rapidly generate a consistent approach. The overall objective of our project was defined as: to develop specifications for the process improvements needed to manage an obstetric emergency (such as PPH) in a woman with suspected or confirmed COVID-19, using rapid, remote consensus-building among multi-professional stakeholder groups. Target audiences included healthcare professionals working in maternity care in UK NHS trusts, including midwives, obstetricians, and managers of maternity services.

### (2) Draft of the proposed process improvement specification

We started by producing Version 1 of a video that showed a simulation of a maternity ward team managing PPH in a woman with suspected or confirmed COVID-19. The video illustrated processes that included: how the team communicated with each other, the woman and her partner; PPE donning and doffing procedures; and use of obstetric-specific procedures (e.g. PPH ‘grab bag’, treatment algorithms) in a COVID-19 context. The processes illustrated were based on: the emerging national guidance on COVID-19 infection prevention and control in a clinical setting (April 2020); clinical guidelines for managing obstetric emergencies such as PPH [e.g. 111, 112]; and ways of working established in one of the safest maternity units of the UK [115, 116]. Though the processes shown thus represented initial reasonable specifications for process improvements to facilitate handling of an obstetric emergency in a COVID-19 scenario, it was also likely that these specifications could be further optimised.

The use of a video format to elicit suggestions for improvement was intended to enhance participant engagement [76, 77], minimise cognitive burden (e.g. not having to study a written manual) [117], and align maternity professionals’ desire to have more COVID-specific e-resources available [104]. The video format was also expected to work well for the study participants using a range of different technologies, including mobile devices [76, 118].

### (3) Participant recruitment strategy

#### Eligibility

We drew on the expertise of three expert groups: maternity teams to provide clinical and practical views; infection prevention and control staff for specialist knowledge on infection guidance; and healthcare human factors specialists for their perspective on the interaction between people and work systems in healthcare settings [21, 119]. The invitation asked potential participants to provide their main professional background, including an “other” option to avoid forcing people into categories that did not suit them. Participants were not further screened for expertise, in order to ensure a rapid response, minimal burden to participants, and maximal inclusivity.

**Fig. 1:**
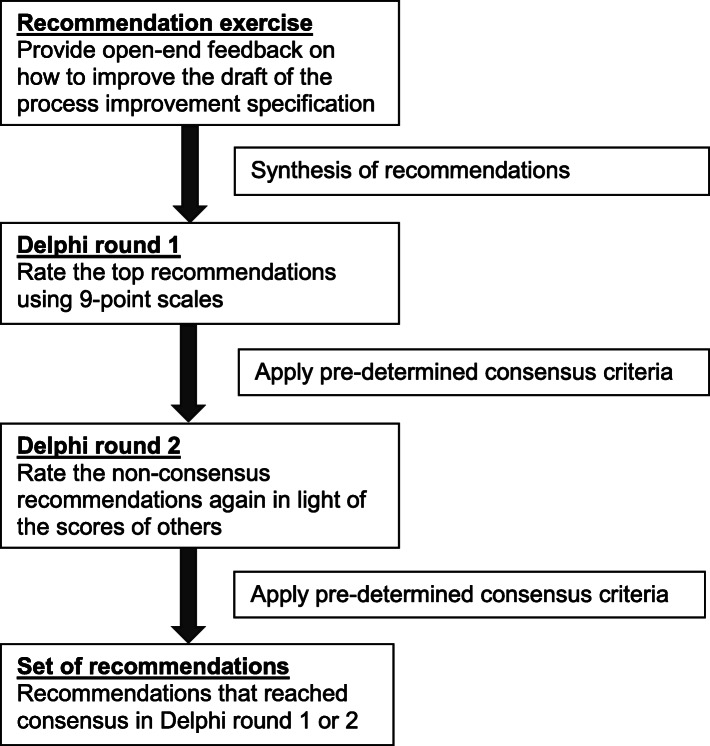
Design of the consensus-building exercise

The consensus-building exercise consisted of a recommendation generation exercise followed by two Delphi rounds (Fig. 1). For the first Delphi round, only those who had taken part in the recommendation exercise and had stated that they were happy to be contacted again were eligible, and for the second Delphi round, only those who had taken part in the first Delphi round were eligible.

#### Sampling and recruitment

We made use of convenience and snowball sampling to rapidly recruit a sample for the three expert groups [120]. Geographical representation was maximised using recruitment conducted through nationwide email networks of specialists in maternity care, infection prevention and control, and healthcare human factors. We anticipated that most participants would engage in response to the email (convenience sampling), while other participants might become involved as a result of colleagues alerting them to the study (snowball sampling). To maximise response rates and minimise attrition bias, reminders were sent through the email networks and Thiscovery, referring to the importance of their (continued) engagement [87, 91].

#### Sample size

We expected that we would require three panels of stakeholders with distinct areas of professional expertise [85], with at least seven experts each, in accordance with conventional recommendations on sample size of an expert panel [61]. In the design of our consensus-building exercise (Fig. 1), we assumed that approximately half of the participants of the initial recommendation exercise would take part in the first Delphi round (50% response rate), with an attrition rate of 20% for the second Delphi round [54]. Accordingly, we aimed to recruit at least 20 stakeholders for each of the three expert groups for the recommendation exercise.

### (4) Remote consensus-building exercise

The recommendation generation exercise and Delphi rounds were designed and then integrated into Thiscovery. All exercises were user tested to optimise participant experience on computer, tablet and smartphone platforms. Data collection and analysis for the consensus-building exercise (including the recommendation generation exercise and Delphi rounds) was completed in 6 weeks.

#### Ethics

Participants registered for an account on Thiscovery after consenting to the platform’s privacy policy and terms of use (https://www.thiscovery.org/register/). Participants confirmed their consent before each new round of the consensus-building exercise. All data was captured and processed without any personal identifiable information. Review by an institutional review board was not applicable, as the project was a consultation and engagement exercise classified as a quality improvement activity [92, 93], in which all of the participants were invited to join the authorship group and to be acknowledged in the project’s outputs.

#### Recommendation exercise

We started with a recommendation generation exercise to inform the subsequent Delphi consensus-building rounds (Fig. 1). This initial exercise consisted of asking participants for their feedback on a draft of good practice in undertaking the process, as illustrated in a video. After seeing the video, participants were asked, using open-ended questions, to draw on their professional expertise to provide recommendations to improve the practice illustrated in relation to: 1) donning PPE; 2) management of the emergency in the context of COVID-19, e.g. use of PPH “grab bag”; 3) doffing PPE; and 4) any other areas.

This first stage of the project – the recommendation exercise – was completed by 105 participants (Table 2). There were 912 responses from 103 participants (two participants did not provide recommendations). Of these 912 responses, 94 were coded as general comments (e.g. “The order of doffing is very important”) or supportive statements (e.g. “The process shown appeared very proficient”) and were excluded from further analysis. The remaining 818 responses were recommendations relating to improvement of practice illustrated in the video, and were synthesised: three analysts worked in parallel on an iterative analysis process of assigning recommendations to pre-defined categories, coding them, re-assigning them to categories more closely fitting with the codes, and merging similar recommendations (Supplement 1). This led to a total of nine categories including 74 synthesised recommendations. Of these, 26 recommendations were identified as the most frequently raised across all participants (Supplement 1).

**Table 2:** Participants in the recommendation generation exercise and Delphi rounds

	Recommendation exercise	Delphi round 1	Delphi round 2
Total (N)	105	71	57
Maternity care (n)	36	23	16
Infection prevention and control (n)	17	7	5
Human factors (n)	52	41	36

A final clinical stakeholder review by authors TD and CW identified two recommendations as only focusing on the video format, rather than on improving the processes illustrated in the video, and were excluded from the consensus-building exercise. This stakeholder review also identified recommendations that could be further combined due to sufficient overlap. We were therefore able to classify the remaining 22 recommendations into five categories (Table 3).

**Table 3:** Recommendations used in the Delphi rounds, median and interquartile (IQR) ratings of participants, and proportion of participants providing a rating of 7, 8 or 9. Legend: Bold numbers indicate recommendations on which consensus was reached. See Supplement 2 for more details

Recommendation to improve processes illustrated in the video	Delphi round 1			Delphi round 2		
	Median	IQR	7–8-9 percentage	Median	IQR	7–8-9 percentage
**Preparation and team roles**						
1. To prevent excessive donning and doffing associated with leaving and re-entering the room, assign someone outside the room to act as a runner. For example, to receive blood samples to send to the lab or to obtain extra equipment if needed.	9	2	**82**			
2. Use role identifiers for staff wearing PPE. For example, staff should wear stickers or laminated photos.	7	3	61	8	2	**81**
3. Have pre-defined key roles for staff during each emergency and allocate these to a specific team at the start of each shift with a buddy system. For example, in the event of a PPH, one staff member’s role would be to prepare the PPH medication in the room.	7	4	59	7	4	63
**Donning of PPE**						
4. Clarify correct sequence for donning gloves and entering room.	9	2	**80**			
5. To avoid contamination, ensure appropriate glove use: wear double gloves, do not open doors with gloved hands, and wear the gloves over the long-sleeved gowns.	8	2	**72**			
6. Perform hand hygiene prior to donning PPE.	9	3	72	9	1	**86**
7. Have a person to assist with donning of PPE if possible. For example, the third team member should receive assistance from the second team member with donning.	7	3	69	8	2	**86**
8. Secure and fix hair away from face to protect hair and face from contamination. For example, use disposable hats, caps or tie hair back.	8	3	68	9	2	**84**
9. A woman with suspected or confirmed COVID-19 should be cared for by staff wearing full protective PPE using a visor and not just a simple mask.	7	4	56	9	4	67
10. Improve gown and apron cover for both the woman and the doctor. For example, tie gown at the side rather than at the back.	6	3	44	7	2	39
11. Avoid giving masks to patients during an emergency (as it compromises breathing and reduces ability to assess).	5	3	32	6	3	47
**Doffing**						
12. Include more time and instruction on the correct doffing order. For example, doff the majority of PPE (gloves and gown) inside the room and doff masks outside of the room.	9	2	**76**			
13. Perform hand hygiene at each stage of the doffing process. For example, perform hand hygiene before or after doffing the gown, before or after doffing gloves, and before doffing the mask or eye protection.	7	4	53	9	3	68
**Layout and design – application of human factors**						
14. Apply human factors principles to the design of the PPE donning station. For example, items in sequence of use, standardised layout.	9	2	**86**			
15. Improve grab bag design. For example, indicate contents, use a box and standardised layout.	9	2	**85**			
16. Provide a clear demarcation of dirty/clean zones to indicate moving in and out of a potential ‘contamination’ zone. For example, mark a red area outside of room for doffing, to ensure potentially contaminated equipment is doffed in a controlled area.	8	2	**80**			
17. Improve bin design to allow easy PPE disposal. For example, wider aperture and a fully opening lid.	8	2	**79**			
**Communication**						
18. Provide instructions to explain correct processes of donning and doffing. For example, a poster on the wall.	9	2	**87**			
19. Provide opportunity for debrief and feedback for the team involved.	9	1	**87**			
20. The importance of communicating with the woman and/or partner should be emphasised. For example, when wearing masks, staff should have awareness of eye contact, tone of voice and body language between the team and towards the woman and partner.	8	2	**80**			
21. Review the design of the algorithm (step-by-step guide). For example, optimise text size and contrast for legibility and provide a hard surface for writing on.	7	2	**76**			
22. Use alternative methods of communication with others outside the room, for example, mobile phones or intercoms.	7	3	61	8	2	68

#### Delphi rounds

All but one of the 105 participants agreed to be contacted again for the Delphi rounds and all 104 were invited. About two-thirds (*n* = 71) of those invited took part in the first Delphi round, of whom 57 took part in the second Delphi round (Table 2). Retention from the first to the second Delphi round was 80% for the total group: 70% for maternity care, 71% for infection prevention and control, and 88% for human factors (Table 2). The risk of attrition bias was low, as ratings in the first Delphi from participants completing both Delphi rounds and from participants who did not respond to the second round were similar.

In the Delphi rounds, the most frequently raised recommendations were presented to participants, with the option to review the video at any point. Participants were asked to rate each recommendation in response to the statement: “This recommendation should be implemented.” A nine-point scale was used with the anchors of “strongly disagree” (rating = 1), “uncertain” (rating = 5), and “strongly agree” (rating = 9). Consensus to implement was defined as > 70% of participants rating a recommendation with a 7, 8 or 9, and < 15% of participants rating it with a 1, 2 or 3, in accordance with core outcome set methodology [54, 121]. An additional criterion we used was an interquartile range ≤ 2, to further validate consensus among the group [98].

Only recommendations that did not reach consensus in the first Delphi round were taken forward to the second [75]. In the second round, the participant was presented with their original rating for each respective recommendation, along with the distribution of ratings from each stakeholder group [122, 123], as shown in an interactive bar chart (see Supplement 2). The participant was asked to consider the ratings of the others, and whether they would like to change their rating or stay with their original rating from the first round.

Of the 22 recommendations rated in the Delphi rounds, 16 reached consensus that they should be implemented (Table 3), with consensus on 12 achieved in the first round and a further four in the second round. The other six recommendations received higher ratings in the second round, but insufficient to reach consensus (only 39 to 68% of participants rated them with a 7, 8 or 9, as shown in Table 3).

Risk of bias due to unequal stakeholder group size was low: analysis showed that consensus across the whole group was similar to that found within the three stakeholder groups, i.e. when each stakeholder group was analysed independently, consensus was reached for an almost identical set of recommendations. For example, “Perform hand hygiene prior to donning PPE” was rated with a 7, 8 or 9 by 74% of maternity care, 71% of infection prevention and control, and 72% of human factor participants (see Supplement 2).

### (5) Final specification of process improvements

The recommendations that reached consensus were reviewed by the project team and used to inform the specification of process improvements for optimised management of obstetric emergencies during the COVID-19 pandemic. These specifications included the processes shown in Version 1 of the video amended based on the consensus-built recommendations (Table 3). A new video (Version 2) to illustrate the specified process improvements was produced [124], along with an infographic [125], and a brief overview of key points [126]. The video and other resources were endorsed by leading organisations who had supported the project, including royal colleges, specialist societies, and quality improvement bodies, and were widely shared. We also shared the resources directly with the participants. We acknowledged the participants’ contribution as collaborators [48].

## Discussion

This article presents a proposed methodological approach aimed at realising a commitment to broad participation, collaboration and consensus-building in process improvement in healthcare. Our case study shows that it is possible to deploy the approach successfully to specify process improvements in an area of pressing need during the COVID-19 pandemic. Subject to further evaluation, this approach has potentially wide application beyond the specific context in which it was tested, and may enable forms of inclusion and collaboration and listening that are otherwise very difficult to do remotely – and do so on a much greater scale. A particular strength of the approach is its ability to support mobilisation of the expertise and ingenuity of people in healthcare systems. This capability can help to enhance the currently limited infrastructure for collaborative building of specifications for process improvement in quality and safety of healthcare. It thus may have potential to address many of the problems of duplication of effort, waste, de-standardisation, and inability to engage sufficiently diverse expertise that currently characterise many quality improvement efforts in healthcare [17, 36].

Our case study showed that the proposed methodological approach can be used successfully to develop specifications for the process improvements needed to ensure high quality care, and may support the production of the kinds of high quality resources that professionals particularly value [104]. The systematic approach to participatory consensus-building that we propose is rich in potential for use in other areas that would benefit from specifying process improvement for clinical scenarios. It may help prevent the characteristic dysfunctions associated with exclusively bottom-up or top-down innovation for quality improvement [16, 17, 22]. Including a large “crowd” of stakeholders can further help mobilise the ingenuity of people in the system (e.g. patients, staff), strengthen the credibility of the consensus-built solution, and enhance feelings of ownership. It may help address lack of access to specific expertise common in locally led, bottom-up approaches [19, 36], and reduce the risks of the excessive focus on compliance associated with top-down approaches [22].

The case study included a wide range of stakeholders as collaborators, thus triangulating the perspectives and expertise of infection prevention and control experts, maternity professionals and healthcare human factors specialists in a way that would not have been possible for a single maternity unit. This blend of expertise was reflected in the participants’ recommendations: many were not tied to the specifics of PPH, but touched on important principles established by infection prevention and human factors specialists [15, 119, 127, 128]. These process improvement specifications are likely to be useful and relevant to multiple clinical communities, particularly those handling emergencies during the pandemic. Using this approach is likely to reduce waste in process improvement, since it not only produces a solution that can be used at scale within the maternity care community, it also generates many core elements of a solution that can be customised for different clinical scenarios outside of maternity. For instance, the relevance of principles of non-technical skills for teams, role assignment, leadership, and ergonomic workspace design when responding to medical emergencies can be generally applicable [127, 129].

A further strength of the approach is that it was possible to undertake this work relatively rapidly – in less than 6 weeks, despite pandemic conditions – and we anticipate that with the formalisation of our framework and with gains in experience, it may be possible for others to replicate the approach in other contexts at an even more rapid pace. Further examples of use cases would help to refine the approach and build a repository that could be used to evaluate it. Over time, the approach may facilitate further work to strengthen the infrastructure for participatory approaches in process improvement, similar to efforts over the last decades for building the infrastructure for clinical guidelines and Core Outcome Set development [1, 2, 54, 72].

Building this infrastructure is critical, because there is an ethical requirement to optimise approaches to process improvement (including specification of improvements) to reduce the risk that people may be avoidably exposed to poorer care associated with sub-optimal processes [36, 130]. Yet results of quality improvement in healthcare are typically mixed [18], suggesting the need to improve how improvement is done. Creating infrastructures for large-scale, collaborative improvement will, of course, require further development, refinement and evaluation of the approach we propose. The participatory ethos on which our approach is built may increase acceptability, uptake and impact of process improvement, but that remains to be tested. It will, for example, be important to study participant experience of taking part in these exercises (e.g. the degree of participation intensity, motivation, engagement, sense of ownership and empowerment [131, 132]), and to assess how far it is possible to reach agreements that stakeholders understand and accept. Evaluation might also examine the potential of using real-time Delphi to avoid having sequential rounds [133–135]. This might improve efficiency, reduce dropout, and minimise participant time, potentially without comprising user experience, inclusivity and robustness of consensus results [136] – though the implications of such an approach for the ability of different groups, such as patients and carers, to participate would require careful evaluation. Other innovations might include application of the principles of gamification to further enhance user experience [137], and provide intuitive, low-burden ways of gaining qualitative feedback on ratings in the Delphi rounds if required [138].

Given our positive experiences in using the approach in the case study, it has potential value in optimising many processes, including, for example, clinical tools and pathways. The approach may also have a role in facilitating implementation of guidance that is written at a high level of generality and requires customisation at local level for particular clinical scenarios, or in assembling relevant elements (e.g. relating to infection control and teamwork) that may be distributed across several guidelines but require integration to achieve patient care goals for specific scenarios [139–141].

In future work, it will important to identify the kinds of applications the approach works best for and where its limits lie. One issue, for example, is that the approach is likely well suited for *specifying* process improvements; locally, organisations will still need to do the work of *implementing* the process improvements, for example through cycles of change and monitoring implementation over time [9, 10]. Another issue concerns for what else the approach could work well for: our case study has used it to specify process improvements, but the broader consensus-building, participatory principles and methods may also have wider potential in, for example, optimising the design of equipment, forms, clinical pathways, and other applications. Evaluation should also establish when application of the approach has created sufficient learning for process improvements in one clinical area such that its learning can be applied to other related clinical scenarios without having to conduct another consensus-building exercise [129, 142, 143]. Finally, there will be an ongoing need to evaluate the process improvements specified through our proposed approach, for examining impacts on implementability, efficiency, staff and service user experience, acceptability, sustainability of change, impact on clinical outcomes, and any unintended consequences. Mixed-methods approaches are likely to be particularly useful in this regard [144].

### Strengths and limitations

The case study illustrated a number of the strengths of our proposed approach. For example, it demonstrated the approach’s effectiveness in engaging a relatively large number of stakeholders as collaborators in a consensus-building exercise to specify process improvements. It also showed the feasibility of rapidly gaining feedback and reaching consensus on the process improvement (< 6 weeks), even during the first peak of the COVID-19 pandemic in the UK, with relatively little attrition (≤; 20%) between the Delphi rounds.

A limitation of the case study is that it did not involve users of maternity services themselves. However, because the methodology facilitates engagement of multiple stakeholder groups [cf. 42, 84], it is potentially well-suited to including service users, patients and carers in future projects. A further limitation is that the three stakeholder groups in our case study were not equal in size. Notwithstanding, the risk of bias appeared to be low: when each stakeholder group was analysed independently, consensus was reached for an almost identical set of recommendations as that determined for the total group. Due to the need to minimise participant burden, we did not collect demographic information that could be used to verify the representativeness of the stakeholders for the population working for or in the NHS. We also had to rely on non-probability sampling techniques that could have created some representation bias. We did, however, ensure that invitations were sent to stakeholder networks across the UK, bolstering our confidence that a representative sample was included. Finally, the case study was conducted during a fast-moving situation where the science (e.g. on infection control) was evolving very rapidly, posing the risk that recommendations made by participants could have been out of date by the time the exercise was completed. This risk was managed by close involvement of clinical expertise from the project team, and by ensuring that process improvements were specified such that they could have enduring relevance (e.g. referring to principles and national guidance on donning and doffing procedures rather than rigidly specifying them).

We were not able to evaluate the implementation or impact of the specified process improvements in the time available or compare our approach with alternative approaches to the specification of process improvements. Both are areas for future development.

## Conclusion

We developed and tested a methodological approach to specifying process improvements that employed a participatory ethos and remote consensus-building methods. The approach was used successfully during pandemic conditions to build consensus among different stakeholder groups on specifying process improvements for managing an obstetric emergency in women with suspected or confirmed COVID-19. The methodological approach has significant potential to support rapid and transparent consensus-building for facilitating process improvement in various healthcare settings using online methods that can be standardised, replicated and scaled when needed, but will require further evaluation.

## Supplementary Information

**Additional file 1: Supplement 1.** Synthesis process of the 912 responses to the recommendation task.

**Additional file 2: Supplement 2.** Interactive bar charts of ratings across the three stakeholder groups as presented to participants in the second Delphi round.

## Data Availability

Queries about the dataset should be directed to the corresponding author.

## Abbreviations

PPH: Post-partum haemorrhage
NHS: National Health Service
PPE: Personal protective equipment
