# Correction to: How to specify healthcare process improvements collaboratively using rapid, remote consensus-building: a frame work and a case study of its application

**DOI:** 10.1186/s12874-021-01345-3

**Published:** 2021-07-29

**Authors:** Jan W. van der Scheer, Matthew Woodward, Akbar Ansari, Tim Draycott, Cathy Winter, Graham Martin, Karolina Kuberska, Natalie Richards, Ruth Kern, Mary Dixon-Woods, Thiscovery Authorship Group, Obstetric Emergency Consensus Authorship Group

**Affiliations:** 1 THIS Institute (The Healthcare Improvement Studies Institute), Departmentof Public Health and Primary Care, University of Cambridge, Cambridge Biomedical Campus, Clifford Allbutt Building, Cambridge, CB2 0AH UK; 2 Department of Translational Health Services, University of Bristol, Bristol, UK; 3 PROMPT Maternity Foundation, Women and Children’s Health, North Bristol NHS Trust, Westbury on Trym, UK

**Correction to: BMC Med Res Methodol 21, 103 (2021)**

**https://doi.org/10.1186/s12874-021-01288-9**

Following publication of the original article [1], the author noticed that:

1. Two names are missing from the acknowledgements section under “Obstetric Emergency Consensus Authorship Group” (“Rachel Corry” and “Celine McKeown”). The list in that section should read:
  Amanda Andrews, Rita Arya, Sarah F. Bell, Denise Chaffer, Andrew Cooney, Rachel Corry, Mair G.P. Davies, Lisa Duffy, Caroline Everden, Theresa Fitzpatrick, Courtney Grant, Mark Hellaby, Tracey A. Herlihey, Sue Hignett, Sarah Hookes, Fran R. Ives, Gyuchan T. Jun, Owen J. Marsh, Tanya R. Matthews, Celine McKeown, Alexandra Merriman, Giulia Miles, Susan Millward, Neil Muchatata, David Newton, Valerie G. Noble, Pamela Page, Vincent Pargade, Sharon P. Pickering, Laura Pickup, Dale Richards, Cerys Scarr, Jyoti Sidhu, James Stevenson, Ben Tipney, Stephen Tipper, Jo Wailling, Susan P. Whalley-Lloyd, Christian Wilhelm, Juliet J. Wood.
2. These two names (“Rachel Corry”, “Celine McKeown”) are also missing from the Authorship Group name and the tagging of them in databases such as Pubmed. The two names need to be added accordingly as well.

The original article has been corrected.
