# Supplementary material for: How to specify healthcare process improvements collaboratively using rapid, remote consensus-building: a framework and a case study of its application
Source: BMC Med Res Methodol. 2021 May 11;21:103. doi: 10.1186/s12874-021-01288-9 (PMC8111055; doi:10.1186/s12874-021-01288-9)
Supplement: Supplementary file 1 — Additional file 1: Supplement 1. Synthesis process of the 912 responses to the recommendation task. [file 12874_2021_1288_MOESM1_ESM.pdf]

## Supplement 1. Synthesis process of the 912 responses to the recommendations task

Three analysts (authors Matt Woodward and Natalie Richards, and analyst Emily Ryen Gloinson) with a background in qualitative analysis and/or healthcare human factors engineering worked in parallel on an iterative qualitative analysis process of assigning recommendations to pre-defined categories, coding them, re-assigning them to categories more closely fitting with the codes, and synthesising similar recommendations. An overview is provided in the figure below. Abductive coding was applied to distil the responses received into a set of recommendations to be presented back to participants for rating. Text was classified as either a recommendation or a comment/observation and only concrete recommendations were taken forward for coding. Recommendations were first grouped into categories that corresponded to the sequence of practice portrayed in the video or to the design of equipment, workstations or physical environment. Two categories were used to include recommendations regarding the content and structure of the video itself. A descriptive code was then assigned to group similar recommendations together. The three analysts reviewed a sample of recommendations as a team to check for consistency of approach, to discuss instances where coding could be ambiguous, and encourage triangulation for the codes applied. The recommendations raised most frequently were provided in a list to clinical stakeholders for review, who for example adjudicated on clarity of phrasing, and on the final categorisation of the recommendations.

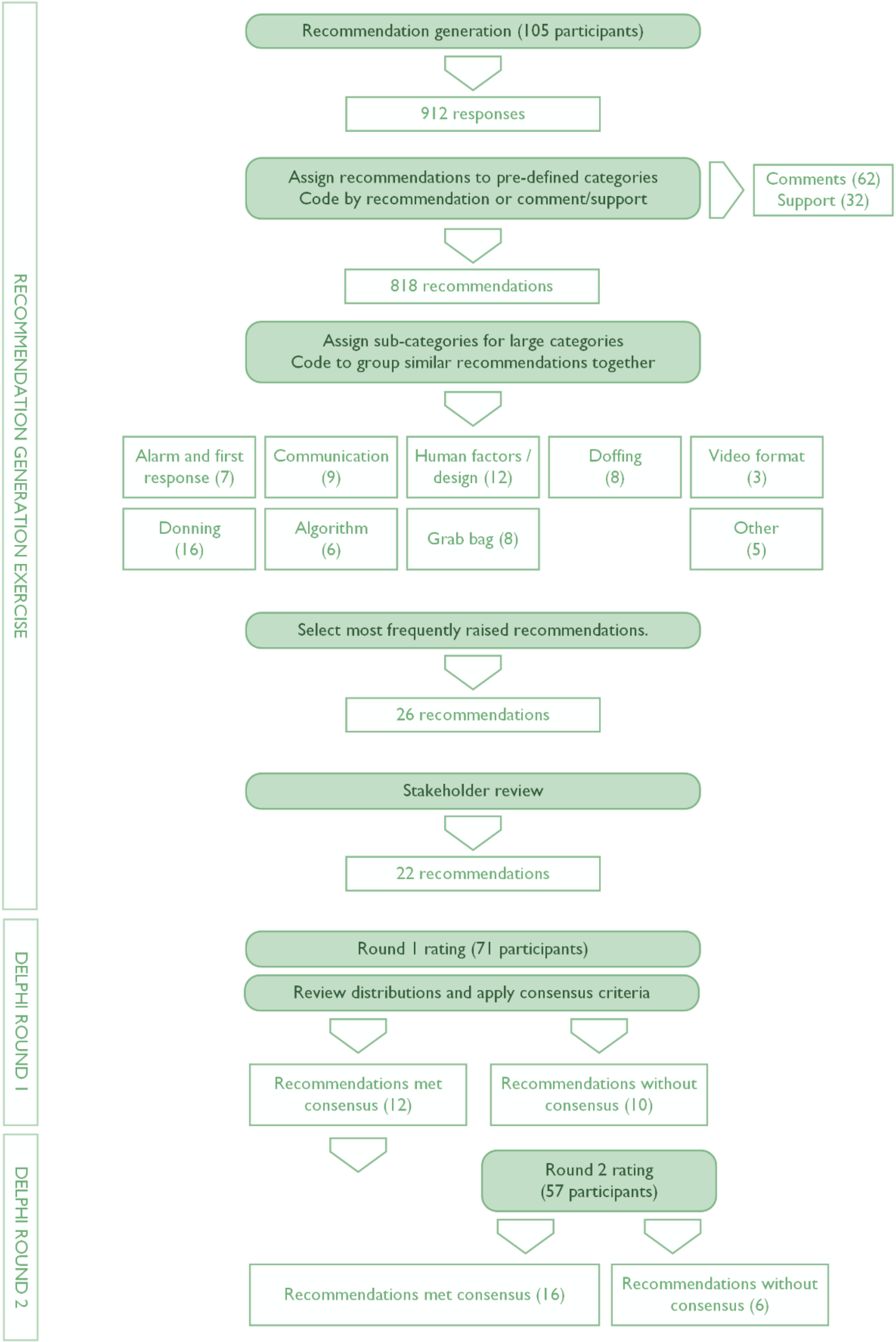
RECOMMENDATION GENERATION EXERCISE
Recommendation generation (105 participants)
912 responses
Assign recommendations to pre-defined categories
Code by recommendation or comment/support
Comments (62)
Support (32)
818 recommendations
Assign sub-categories for large categories
Code to group similar recommendations together
Alarm and first response (7)
Communication (9)
Human factors / design (12)
Doffing (8)
Video format (3)
Donning (16)
Algorithm (6)
Grab bag (8)
Other (5)
Select most frequently raised recommendations.
26 recommendations
Stakeholder review
22 recommendations
DELPHI ROUND 1
Round 1 rating (71 participants)
Review distributions and apply consensus criteria
Recommendations met consensus (12)
Recommendations without consensus (10)
DELPHI ROUND 2
Round 2 rating (57 participants)
Recommendations met consensus (16)
Recommendations without consensus (6)
