# Supplementary material for: How to specify healthcare process improvements collaboratively using rapid, remote consensus-building: a framework and a case study of its application
Source: BMC Med Res Methodol. 2021 May 11;21:103. doi: 10.1186/s12874-021-01288-9 (PMC8111055; doi:10.1186/s12874-021-01288-9)
Supplement: Supplementary file 2 — Additional file 2: Supplement 2. Interactive bar charts of ratings across the three stakeholder groups as presented to participants in the second Delphi round. [file 12874_2021_1288_MOESM2_ESM.pdf]

Supplement 2. Interactive bar charts of ratings across the three stakeholder groups as presented to participants in the second Delphi round

In the second Delphi round, participants were presented with the ratings of the recommendations that did not reach consensus in the first Delphi round. This included their original rating, along with the distribution of ratings from each stakeholder group as shown in an interactive bar chart.

These interactive bar charts are shown here: https://pph-charts.carrd.co/
